# BSS-YOLO: honey peach ripeness detection in complex orchard scenes under simulated adverse-weather conditions and its implications for fruit industry management

**DOI:** 10.3389/fpls.2026.1929998

**Published:** 2026-10-06

**Authors:** Junwei Tian, Xiaoye Zhang, Feipeng Gao, Jie Yang, Cong Zhou

**Affiliations:** 1 College of Artificial Intelligence, Zhejiang A&F University, Hangzhou, China; 2 School of Information and Communication Engineering, Hainan University, Haikou, China; 3 Jiyang College, Zhejiang A&F University, Zhuji, China; 4 College of Economics and Management, Zhejiang A&F University, Hangzhou, China

**Keywords:** deep learning, honey peach, operational efficiency, ripeness perception, YOLO

## Abstract

Accurate honey peach ripeness detection in natural orchards remains challenging because complex orchard factors, including strong illumination, fruit occlusion and overlap, target-scale variation, and weather-related image degradation such as haze and rainfall, can degrade image quality and weaken the visual discrimination among different ripeness stages. To improve ripeness detection under such interference, this study proposes an improved YOLOv12s-based method termed BiFPN–SCSA–SD-DIoU YOLO (BSS-YOLO). First, a multi-meteorological honey peach ripeness data set is constructed by combining field-collected orchard images with physics-driven haze and rainfall simulation, providing diverse weather-degraded samples for model training and evaluation. Second, a Spatial-Channel Synergistic Attention (SCSA) mechanism is embedded into the backbone network to strengthen the representation of ripeness-related regions while suppressing weather-induced background interference. Third, a Weighted Bidirectional Feature Pyramid Network (BiFPN) is introduced into the neck to enhance adaptive multi-scale feature fusion for peaches with different sizes and occlusion levels. Furthermore, a scale-dynamic distance intersection over union (SD-DIoU) loss function is adopted to improve bounding box regression stability for multi-scale targets under the evaluated simulated fog- and rain-degraded conditions. Experimental results show that BSS-YOLO achieves 93.4% mAP50 and 85.0% mAP50–95 on the constructed multi-meteorological dataset containing original sunny-weather images and simulated fog and rain images, with only 8.57 million parameters and an inference speed of 63.5 FPS. Compared with the YOLOv12s baseline, the proposed method improves mAP50 and mAP50–95 by 2.0 and 1.9 percentage points, respectively, while reducing the parameter count from 9.07M to 8.57M. Moreover, compared with the recently proposed peach-related EMA-YOLO model, BSS-YOLO achieves higher mAP50 and mAP50-95, showing higher detection accuracy on the evaluated dataset. These results indicate that the proposed method has potential for honey peach ripeness monitoring under the evaluated weather-related degradation conditions, and may provide auxiliary support for automated fruit sorting, harvest-related decision-making, and operational efficiency improvement in fruit industry management.

## 1 Introduction

Honey peaches are a fruit that combines both health and economic value, with their primary cultivation areas concentrated in Asia and Europe. Against the backdrop of rapid advancements in smart agriculture and fruit industry management, accurate assessment of fruit ripeness can provide useful information for harvest-related decision-making, support automated fruit sorting, and enhance orchard operational efficiency. Traditional methods relying on human visual inspection for ripeness assessment are not only time-consuming and labor-intensive but also inefficient. Compounded by the growing shortage of rural labor and an aging population, rising labor costs have become a bottleneck constraining industry development (Li et al., 2024). Furthermore, such methods are susceptible to interference from subjective experience and external environmental factors, making it difficult to consistently assess fruit ripeness and support harvest-related decisions, coordinate orchard operations, and reduce resource wastage.

To automate this process, early fruit detection primarily relied on traditional machine vision and machine learning methods (Jiménez et al., 2000; Gongal et al., 2015). These approaches involve the manual design of features such as color and texture, combined with classifiers like Support Vector Machines (SVM) to achieve recognition. However, such methods are heavily dependent on stable lighting conditions and are prone to failure under shadow, backlight, or changing weather conditions. Furthermore, they exhibit insufficient generalization capabilities for overlapping fruits and complex backgrounds, making it difficult to meet the high-precision detection demands of modern agriculture.

To overcome these limitations, in recent years, with the continuous advancement of artificial intelligence and the progress of computer vision technology, object detection models based on convolutional neural networks (CNNs) have matured significantly, demonstrating immense potential particularly in agricultural fruit tree applications. Among these, the YOLO (You Only Look Once) series, as a representative of single-stage detection algorithms, has rapidly gained widespread attention in this field due to its high accuracy, fast recognition efficiency, and lightweight deployment advantages (MacEachern et al., 2023).

Considering the rapid progress of deep learning, CNN-based object detection methods have mainly developed along two-stage and single-stage paradigms. Two-stage detectors, such as Faster R-CNN (Ren et al., 2017), generally provide strong detection accuracy but involve relatively high computational costs and latency, whereas single-stage detectors place greater emphasis on real-time efficiency. SSD (Liu et al., 2016) improves multi-scale detection through predictions from multiple feature maps, while the YOLO series has become widely used because of its favorable balance between detection accuracy and inference efficiency. From YOLOv3 (Redmon and Farhadi, 2018) to more recent versions, the YOLO family has continued to evolve toward more efficient feature extraction, simplified detection pipelines, and improved feature representation. Recent models, including YOLOv8 (Yaseen, 2024), YOLOv10 (Wang et al., 2024), YOLOv11 (Khanam and Hussain, 2024) and YOLOv12 (Alif and Hussain, 2025; Khanam and Hussain, 2025; Tian et al., 2025) further reflect this trend, with continued efforts to balance detection accuracy, computational efficiency, and real-time performance. In parallel, Transformer-based end-to-end detectors have also attracted increasing attention. Zhao et al. (2024) introduces an efficient hybrid encoder and uncertainty-minimal query selection strategy, enabling real-time end-to-end object detection while reducing dependence on NMS post-processing. In recent years, the enhanced YOLO algorithms have garnered significant attention for their application in the domains of fruit and disease detection, showcasing remarkable advancements in addressing natural environmental disturbances. Related studies on other fruit and nut vision tasks have also explored complementary directions, including YOLO-based ripeness detection, lightweight CNN design, attention mechanisms, and metaheuristic feature selection. Yurdakul et al. (2026) developed a public strawberry ripeness dataset and comparatively evaluated multiple YOLOv8, YOLOv9, and YOLO11 variants, providing a recent reference for YOLO-based fruit ripeness detection.

To address the challenges posed by occlusion and small targets, Xu et al. (2024) presented EMA-YOLO, which incorporated an EMA attention mechanism and demonstrated a notable enhancement in the detection rate of unripe yellow honey peaches in conditions of leaf and branch occlusion. Subsequently, Wang et al. (2025) and Yao et al. (2025) proposed YOLO-ALW and WTAD-YOLO, respectively, which significantly bolstered the detection accuracy of pepper and tomato diseases by fortifying feature fusion networks. In the domain of lightweight deployment, Zhu et al. (2024) embarked on the implementation of an oil tea fruit ripeness detection system, underpinned by an enhanced YOLOv7-tiny model. In a parallel vein, Chen et al. (2025) proposed the integration of FasterNet with Wise-IoU, giving rise to FEW-YOLO, an approach tailored for the optimization of goji berry detection. Moreover, Yu et al. (2025) and Cuong et al. (2022) also served to validate the effectiveness of lightweight models in strawberry and pineapple tasks, thereby providing further confirmation that pruning or introducing modules such as GhostNet and MobileNet can substantially enhance YOLO’s applicability on embedded devices.

Similar lightweight and attention-based strategies have also been investigated in agricultural product classification. Yurdakul et al. (2024a) designed a lightweight CNN for almond variety classification using genetic optimization, while Yurdakul et al. (2025) incorporated attention mechanisms into CNN-based chestnut classification to enhance feature representation. In addition, metaheuristic optimization has been explored for feature selection in agricultural vision tasks: Yurdakul et al. (2024c) employed the Flower Pollination Algorithm for almond classification, whereas Yurdakul et al. (2024b) investigated Harris Hawks Optimization-based feature selection for chestnut classification.

In specific fruit studies, Zhao et al. (2023) constructed a dataset for honey peaches and proposed a single-stage instance segmentation model, effectively mitigating lighting variations and fruit adhesion issues. Huang et al. (2025) sought to enhance the feature extraction capabilities of ORD-YOLO, with the objective of improving citrus ripeness recognition in complex backgrounds. Regarding adverse weather conditions, Li et al. (2025) employed dynamic snake convolutions with DSF-YOLO to enhance small object detection in rain and fog, thus providing pivotal insights for visual detection in agricultural settings under all weather conditions.

Despite the advances in fruit detection and lightweight deployment achieved in existing research, significant shortcomings persist in honey peach ripeness detection under complex meteorological conditions. Primarily, there is a lack of data from diverse weather scenarios. Existing honey peach visual inspection studies predominantly focus on sunny days or conventional natural lighting conditions, whereas adverse meteorological factors, such as low-contrast imaging during foggy days and rainwater obstruction and reflection during rainy days, have not been sufficiently investigated. This limitation leads to datasets that are inadequate for capturing real-world orchard operations across diverse weather conditions, consequently resulting in insufficient validation of model robustness. Additionally, feature extraction capabilities are constrained under extreme environmental conditions. Under low visibility or rainy conditions, fruit color, texture, and boundary information are susceptible to substantial degradation, making it difficult for conventional models to accurately determine fruit ripeness.

To address honey peach ripeness detection under weather-related visual degradation, this study proposes BSS-YOLO, an improved YOLOv12s-based detection method evaluated using field-collected sunny orchard images and physics-simulated fog and rain images.

1. A multi-meteorological honey peach ripeness dataset is constructed by combining field-collected sunny orchard images with physics-simulated fog and rain images. The dataset provides controlled and reproducible samples for evaluating ripeness detection under sunny and simulated adverse-weather conditions.
2. The SCSA module proposed by (Si et al., 2025) is incorporated into the YOLOv12s backbone to strengthen joint spatial–channel feature representation, while Weighted BiFPN is introduced into the neck to improve learnable weighted multi-scale feature fusion. In addition, the SD-DIoU loss is adopted to improve bounding-box regression stability for peach targets at different scales.
3. Experiments on the constructed dataset demonstrate that the proposed method achieves improved detection performance compared with the YOLOv12s baseline under the evaluated conditions, including simulated fog and rain degradation.
4. The operational implications of BSS-YOLO are discussed for fruit industry management. By providing visual ripeness information under the evaluated conditions, the proposed method may provide auxiliary support for harvest-related decision-making, automated sorting, and the allocation of labor and equipment resources.

## 2 Materials and methods

### 2.1 Image dataset acquisition

The experimental data were collected at the Zhongcun Fresh Fruit Farm base in Xihu District, Hangzhou City, Zhejiang Province. The geographic location of the field data collection site is shown in **Figure 1**. This region features a subtropical monsoon climate with abundant sunlight and rainfall coinciding with the warm season, creating ideal conditions for honey peach cultivation. Data collection focused on the period from early to mid-June (covering the time between the Grain in Ear and Summer Solstice solar terms), encompassing the entire growth stage of honey peaches from color change to maturity. Image capture was performed utilizing an IQOO Neo15 smartphone, operating at a resolution of 4096 × 3072 pixels. No manual spatial cropping was applied to the original images during dataset construction. The complete images were retained and processed to the network input resolution of 640 × 640 using the standard Ultralytics preprocessing pipeline, with aspect-ratio-preserving resizing and padding before network input. All original field images were collected under sunny-weather conditions, including scenes affected by strong illumination and backlighting. No naturally occurring foggy or rainy images were included in the original dataset; these adverse-weather conditions were subsequently introduced through simulation-based meteorological degradation after dataset partitioning. Shooting distances were maintained between 30–100 cm. This approach is consistent with the findings of previous studies conducted by Liu and Yin (2023) and Li et al. (2024). The collected images covered diverse orchard scenes, including single and multiple fruit targets, branch or leaf occlusion, and fruit overlap, thereby providing varied visual conditions for subsequent model training and evaluation. After routine image-quality checking, a total of 2,000 original field-collected images were retained for subsequent annotation and dataset construction.

**Figure 1:**
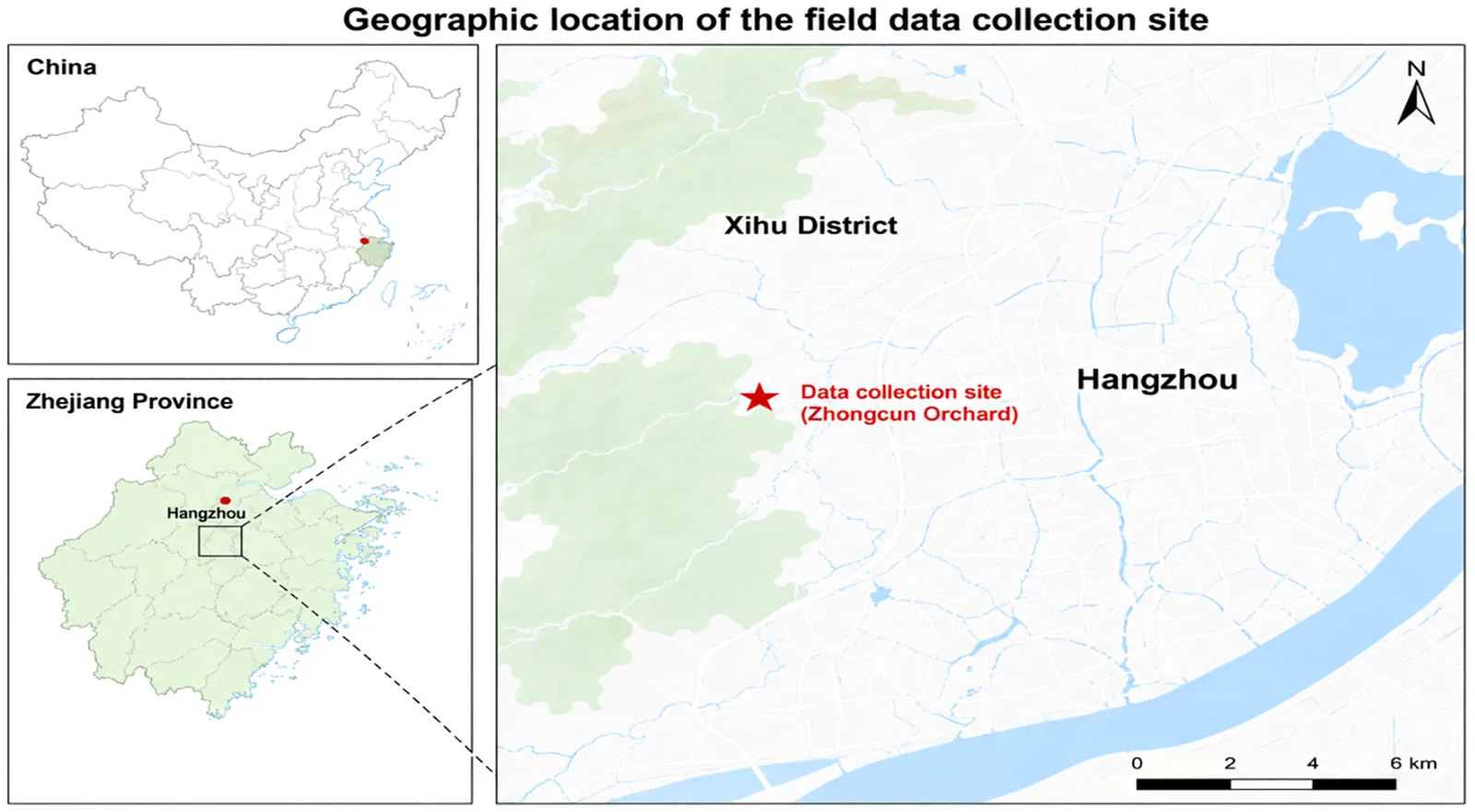
Geographic location of the field data collection site in Xihu District, Hangzhou City, Zhejiang Province, China.

### 2.2 Data creation and preprocessing

#### 2.2.1 Standards for the classification of fruit maturity

This study refers to the agricultural industry standards of the People’s Republic of China, Grade and Specification for honey peaches (NY/T 586-2002) and Technical Code for Post-harvest Handling of honey peaches (NY/T 866-2004), while also incorporating the classification strategies proposed by Xu et al. (2024) and Zhao et al. (2023) for honey peach ripeness detection. Accurate assessment of visual ripeness is important for supporting harvest management and reducing quality losses associated with premature or delayed harvesting. Accordingly, the ripeness categories in this study were defined primarily according to visually observable peel-color characteristics and were divided into three levels:

Unripe (UR): The fruit’s exterior is predominantly greenish, showing no significant signs of color transition on the surface.

Half-ripe (HR): Color transition begins at the fruit apex or the light-exposed surface, exhibiting hues of light green, pale yellow, or pinkish streaks.

Ripe (R): The fruit surface is predominantly red. A red-color proportion of approximately 75% was used as an operational reference during visual annotation.

The above color-based criteria were used for image-based visual ripeness categorization and were not treated as direct measurements of physiological maturity. Representative examples of the three ripeness grades are shown in **Figure 2** to provide a visual illustration of the classification criteria.

**Figure 2:**
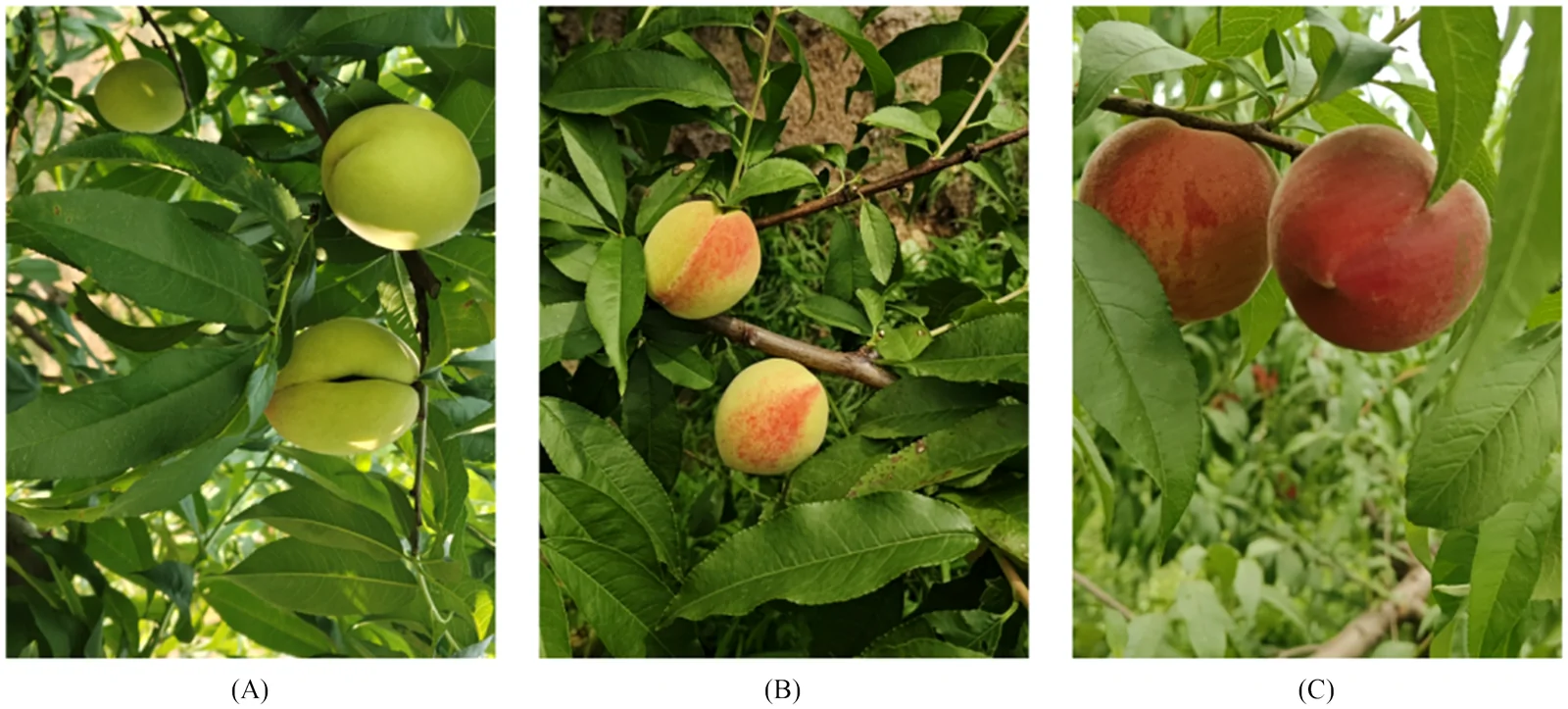
Representative images of the three honey peach ripeness grades: **(A)** unripe (UR), **(B)** half-ripe (HR), and **(C)** ripe (R).

#### 2.2.2 Dataset annotation and partitioning

The original images were manually annotated using LabelImg, and the resulting labels were saved in YOLO (.txt) format. The annotation criteria described in Section 2.2.1 were applied consistently throughout the dataset. The initial annotations were completed by one researcher and subsequently reviewed by the supervisor and other members of the research team. Targets with more than 75% occlusion, severely blurred boundaries, or substantially incomplete contours were excluded from annotation. Accordingly, these excluded cases fall outside the quantitative evaluation range of the present study. These exclusion criteria were applied to maintain the integrity and quality of the annotated samples. This rigorous approach is undertaken to guarantee the integrity and high-quality of the training samples. Following the completion of the annotation process, the 2,000 original field-collected images were first divided into training, validation, and test sets at a ratio of 8:1:1, resulting in 1,600, 200, and 200 images, respectively. Importantly, dataset partitioning was completed before meteorological simulation. Therefore, all simulated weather variants derived from a given original image remained within the same subset, preventing derivatives of the same source image from appearing across the training, validation, and test sets. This approach aligns with the data allocation strategies proposed by Zhang et al. (2024) and Assunção et al. (2022), providing standardized data to support model training and performance evaluation.

In terms of instance distribution, a total of 4,921 valid honey peach instances (bounding boxes) were obtained from the original 2,000 images. Specifically, the dataset contains 1,647 instances for the Unripe class, 1,860 for the Half-ripe class, and 1,414 for the Ripe class. This relatively balanced class distribution effectively mitigates the risk of class imbalance during the model training phase.

#### 2.2.3 Simulated meteorological data augmentation

To increase the diversity of weather-degraded samples, offline fog and rain augmentation was applied after the original dataset had been partitioned into training, validation, and test sets. The augmentation procedure was based on simulation strategies adapted from previous studies (Liu and Yin, 2023; Li et al., 2025). For each original sunny-weather orchard image, one simulated fog image and one simulated rain image were generated within the corresponding dataset subset. Consequently, the training set increased from 1,600 original images to 4,800 images, while the validation and test sets each increased from 200 to 600 images. Thus, within each subset, the ratio of original sunny-weather images, simulated fog images, and simulated rain images was 1:1:1. The detailed composition of the augmented dataset is summarized in **Table 1**.

**Table 1:** Composition of the original and simulated weather data in each dataset split.

Dataset split	Original sunny-weather images	Simulated fog images	Simulated rain images	Total
Training	1,600	1,600	1,600	4,800
Validation	200	200	200	600
Test	200	200	200	600
Total	2,000	2,000	2,000	6,000

To generate controllable weather-related image degradation, the augmentation procedure was further designed with reference to physics-based image degradation studies (Sakaridis et al., 2018; Hendrycks and Dietterich, 2019). Rather than using only arbitrary pixel-level perturbations, the procedure incorporates depth-dependent atmospheric attenuation and synthetic rain effects to produce controlled fog- and rain-degraded samples. These simulated samples are used in this study as reproducible adverse-weather degradation conditions and are not regarded as substitutes for naturally captured foggy or rainy orchard images.

### 2.3 Model construction

To address honey peach ripeness detection under complex orchard imagery and weather-related visual degradation, this paper proposes BSS-YOLO, an improved YOLOv12s-based model evaluated using field-collected sunny images and physics-simulated fog and rain images. YOLOv12s is adopted as the baseline model. Based on this baseline, BSS-YOLO improves the model from three aspects: data construction, feature modeling, and regression optimization. To provide a visual illustration of the overall network architecture of the proposed model and the positional relationships among its enhanced modules, **Figure 3** presents the schematic diagram of BSS-YOLO. Compared with the baseline YOLOv12s structure, the proposed model introduces improvements at the data, backbone, neck, and loss-function levels. Specifically, at the data level, a physics-driven meteorological degradation augmentation strategy is introduced to increase training data coverage under simulated fog- and rain-degradation conditions. The second aspect of the study involves the integration of a spatial–channel synergistic attention (SCSA) mechanism into the backbone network, with the aim of enhancing key feature representation. Concurrently, the neck network benefits from the fusion of a weighted bidirectional feature pyramid network (BiFPN), which works to improve multi-scale feature integration. Finally, the introduction of a scale-dynamically adjusted SD-DIoU loss function serves to enhance bounding box regression stability for targets at different scales.

**Figure 3:**
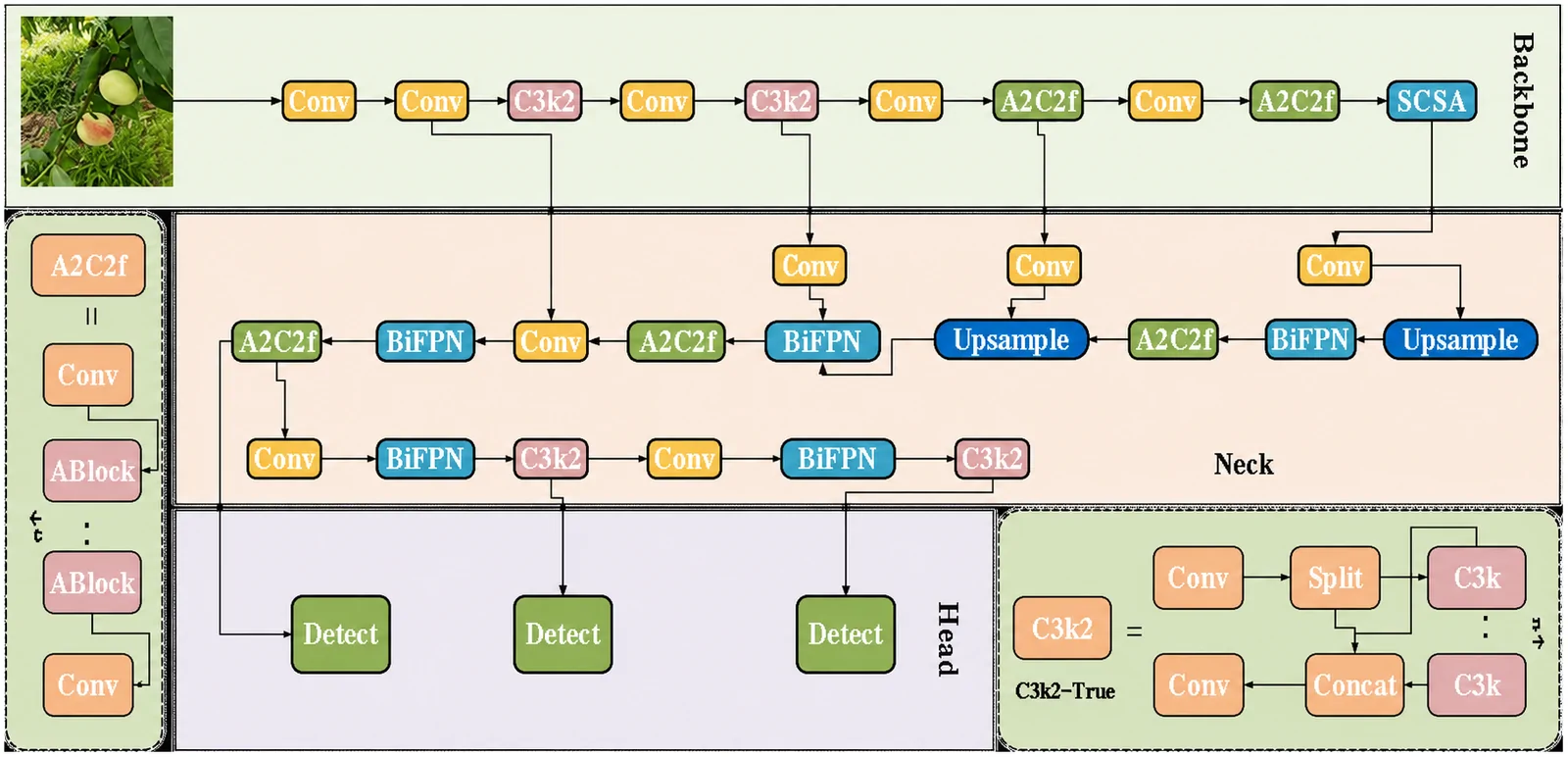
Flowchart of BSS-YOLO. The part marked by the Light blue box is the improved part of this model.

#### 2.3.1 A physics-inspired strategy for meteorological degradation augmentation

To generate controllable fog- and rain-degraded samples, a physics-inspired meteorological degradation augmentation strategy was adopted. The physical formulations are used to introduce depth-dependent and controllable image degradation rather than to quantitatively reconstruct field-calibrated meteorological conditions.

For fog augmentation, based on the classical atmospheric scattering model (He et al., 2009), the formation of hazy images can be expressed as (Equation 1):

(1) $$I(x)=J(x)e^{-\beta d_{n}(x)}+A(1-e^{-\beta d_{n}(x)})$$

where J(x) denotes the original clear image, I(x) denotes the synthesized foggy image, β denotes the effective attenuation coefficient, A represents the atmospheric-light color vector, and dn(x) denotes the normalized relative-depth map.

The relative scene depth was estimated using the pretrained MiDaS DPT-Hybrid monocular depth model. Since the model predicts relative inverse depth rather than metric scene distance, the predicted map was clipped at the 2nd and 98th percentiles, followed by min–max normalization and inversion. A 5 × 5 Gaussian filter was subsequently applied to obtain a spatially smoothed normalized relative-depth map ${\text{d}}_{\text{n}}(\text{x})\in [0,1]$, where larger values correspond to relatively more distant regions. Atmospheric light A was estimated as the mean color of the brightest 0.1% pixels in the input image.

Although the augmentation implementation allows different degradation levels to be specified, only the medium fog setting was used in this study. The effective attenuation coefficient was therefore fixed at β=2.20, rather than being sampled among different fog-intensity categories. The depth-dependent formulation introduces spatially varying attenuation across regions with different relative depths rather than applying spatially uniform fog to the whole image, consistent with the general image-degradation formulations discussed by He et al. (2009).

After atmospheric scattering, a mild global veil with a blending strength uniformly sampled from 0.16–0.24 was applied. Saturation and brightness were subsequently scaled by 0.78 and 0.72, respectively, followed by a contrast factor of 0.88, an intensity offset of −10, and 5 × 5 Gaussian smoothing.

For rain augmentation, the degradation procedure was divided into macro-scale background attenuation and a synthetic rain-streak component, following the general meteorological degradation formulation used by Halder et al. (2019). The macro-scale attenuation is expressed as (Equation 2):

(2) $$L_{ext}(x)=e^{-0.312R^{0.67}d_{eff}(x)}$$

where $L_{\text{ext}}(x)$ denotes the macro-scale attenuation factor, *R* is the controllable rainfall-rate parameter, and $d_{\text{eff}}(x)$ represents the effective depth used in the attenuation calculation. Since the monocular depth estimator provides relative rather than metric depth, $d_{\text{eff}}(x)$ was obtained from the normalized relative depth as shown in Equation 3:

(3) $$d_{eff}(x)=\lambda d_{n}(x),\;\lambda =0.12$$

where $d_{n}(x)$ denotes the normalized relative depth map, and λ=0.12 is the scaling factor used to convert the relative depth into the effective depth. The macro-scale attenuated background image is then calculated using Equation 4:

(4) $$I_{att}(x)=I_{bg}(x)L_{ext}(x)$$

where $I_{\text{att}}(x)$ denotes the macro-scale attenuated background image, and $I_{\text{bg}}(x)$ denotes the original background image.

Only the medium rain setting was used in this study. For each augmented image, R was uniformly sampled from 5–15 mm/h, while the rain-streak density was uniformly sampled from 0.40–0.50. These parameters are used as controllable augmentation settings rather than field-calibrated measurements of actual rainfall conditions.

The synthetic rain-streak layer S(x) was constructed using randomly distributed elongated streaks. The principal streak direction was uniformly sampled from 10° to 28°, the streak length ranged from 45 to 72 pixels, and the thickness ranged from 4 to 5 pixels. Directional motion blur and a spatial random mask were subsequently applied to reduce overly uniform streak patterns.

This treatment retains the rain-streak simulation concept described in previous rain-imaging studies, including (Garg and Nayar, 2006; He et al., 2009; Halder et al., 2019), without interpreting the synthetic streak layer as a direct reconstruction of natural rainfall.

The rain-streak layer was finally combined with the macro-scale attenuated background using (Equation 5):

(5) $$I_{rain}(x)=\alpha S(x)+(1-\alpha )I_{att}(x)$$

Where $I_{\text{rain}}(x)$ denotes the synthesized rainy image and α=0.26 controls the contribution of the rain-streak layer.

After blending, saturation and brightness were scaled by 0.72 and 0.65, respectively, followed by a gamma adjustment of 1.20 and a 6% cool-gray blending operation to obtain the final rainy appearance. The main parameter settings used in the meteorological degradation augmentation procedure are summarized in **Table 2**.

**Table 2:** Main parameter settings used for the physics-inspired meteorological degradation augmentation.

Component	Parameter	Setting used in this study
Depth estimation	Network	MiDaS DPT-Hybrid
Depth processing	Normalization	2nd–98th percentile clipping → min–max normalization → inversion
Depth processing	Spatial smoothing	5 × 5 Gaussian filter
Fog	Intensity level	Medium only
Fog	β	2.20
Fog	Atmospheric light A	Mean color of brightest 0.1% pixels
Fog	Global veil	U(0.16, 0.24)
Rain	Intensity level	Medium only
Rain	R	U(5, 15) mm/h
Rain	λ	0.12
Rain	Streak density	U(0.40, 0.50)
Rain	α	0.26
Rain streak	Direction	U(10°, 28°)
Rain streak	Length	45–72 pixels
Rain streak	Thickness	4–5 pixels

#### 2.3.2 SCSA collaborative attention in the backbone network

Although physics-driven data augmentation expands the diversity of weather-degraded samples, the simulated fog and rain degradation, together with strong illumination in the field-collected images, increases the difficulty of extracting discriminative features for honey peach ripeness recognition. Existing attention mechanisms have been widely used to model spatial or channel information (Woo et al., 2018; Hu et al., 2020; Hou et al., 2021). In this study, the SCSA module proposed by Si et al. (2025) is incorporated into the YOLOv12s backbone. SCSA was selected because its spatial–channel synergistic design is consistent with the feature representation requirements of honey peach ripeness detection. Specifically, spatial information related to fruit regions and structures, together with channel-related appearance cues such as color information, is relevant to distinguishing visually similar ripeness stages. The original SCSA study also evaluated SCSA alongside commonly used attention mechanisms, including CBAM and Coordinate Attention, on standard benchmark tasks (Si et al., 2025), providing additional reference for the selection of SCSA in this study.

SCSA consists of Shared Multi-Semantic Spatial Attention (SMSA) and Progressive Channel-wise Self-Attention (PCSA), which are connected sequentially. SMSA extracts multi-semantic spatial information through multi-scale operations and provides spatial priors for subsequent channel modeling, while PCSA further refines channel relationships based on the spatially modulated features (Si et al., 2025). The overall form of the module can be expressed as (Equation 6):

(6) SCSA(X)=PCSA(SMSA(X))

In this model, X denotes the input features. SMSA captures structural and regional information at multiple semantic scales, thereby providing spatial guidance for subsequent channel modeling. Group Normalization is employed within SMSA to normalize the divided sub-features and improve the stability of feature processing (Wu and He, 2020). Based on the spatial information provided by SMSA, PCSA progressively models inter-channel relationships through channel-wise self-attention, strengthening the joint representation of spatial and channel information. A schematic illustration of the SCSA module is shown in **Figure 4**.

**Figure 4:**
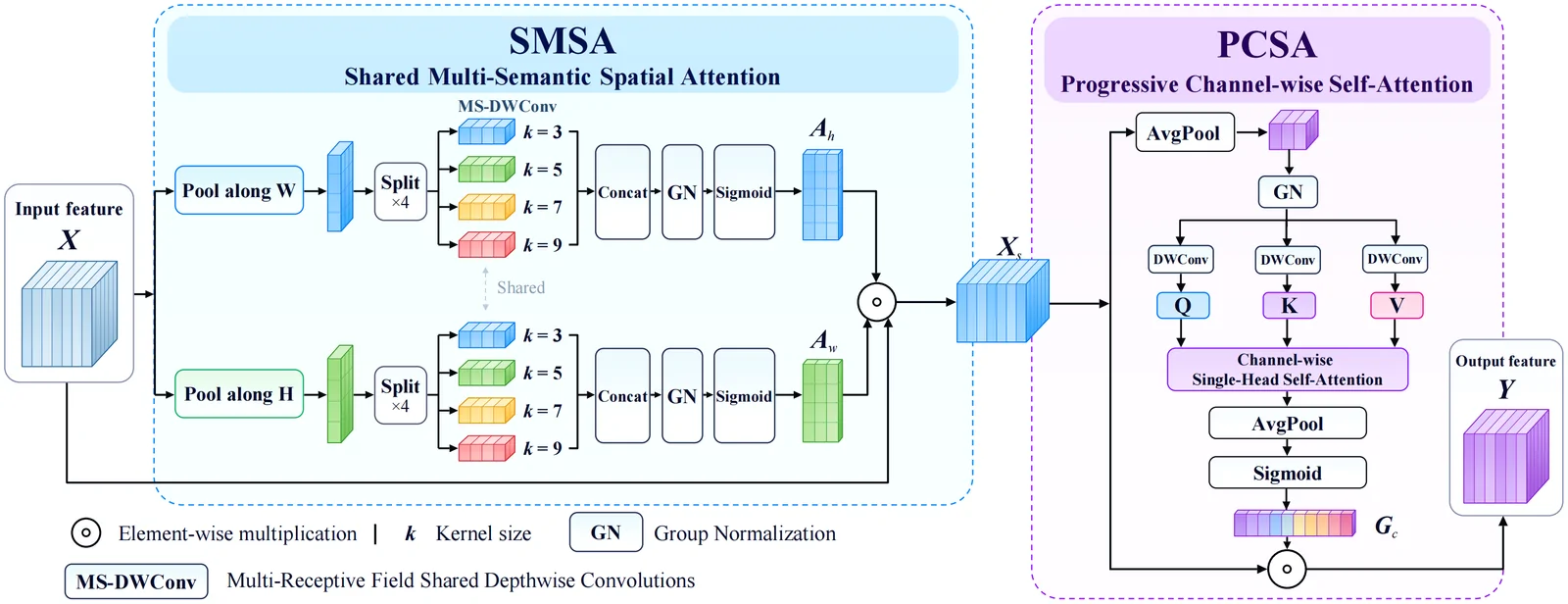
Schematic illustration of the SCSA module, including Shared Multi-Semantic Spatial Attention(SMSA) and Progressive Channel-wise Self-Attention (PCSA). B denotes the batch size, C denotes the number of channels, and H and W denote the height and width of the feature maps, respectively.

By jointly modeling spatial and channel information, SCSA strengthens feature representation in the YOLOv12s backbone and provides a feature basis for the subsequent multi-scale feature fusion stage.

#### 2.3.3 Enhancement based on weighted BiFPN

Honey peaches cultivated in natural orchards encounter significant challenges in maturity detection due to complex visual interference, including intense illumination, simulated fog and rain degradation, fruit occlusion, and substantial target-scale variation. Despite the utilization of spatial–channel synergistic attention (SCSA) by the backbone network to achieve noise disentanglement and feature refinement during the feature extraction stage, honey peach maturity detection remains hindered by severe scale variations and multi-scale information from different feature pyramid levels requires further integration. This study optimizes the neck component of YOLOv12 by integrating a weighted BiFPN module, with the objective of alleviating the conflicts caused by scale variations and environmental noise that critically affect honey peach detection accuracy under the evaluated image-degradation conditions. Conventional approaches, such as Feature Pyramid Networks (FPN) (Lin et al., 2017) or PANet (Liu et al., 2018), adopt fixed aggregation paths and assign equal weights to multi-scale feature contributions. However, features from different pyramid levels have different spatial resolutions and semantic characteristics, and their contributions to the fused representation may therefore be unequal. Simple equal-weight fusion may not fully exploit the complementary information provided by multi-scale features (Liu et al., 2019). Therefore, a learnable weighted fusion mechanism is introduced to improve multi-scale feature utilization.

In order to mitigate feature conflicts, the present study draws inspiration from the design philosophy of EfficientDet (Tan et al., 2020). By introducing bidirectional feature propagation paths—top-down and bottom-up—it enhances information exchange between features at different scales. Compared to traditional pyramid structures, BiFPN reduces computational overhead while improving feature utilization efficiency. This is achieved by removing redundant nodes with only single inputs and introducing skip connections between features at the same scale. The standard Weighted BiFPN was adopted because it provides bidirectional cross-scale connections and learnable weighted fusion within a relatively simple and well-established structure. In this study, BiFPN is used to improve multi-scale feature interaction within YOLOv12s rather than to redesign the feature-pyramid architecture. Although more recent feature-pyramid variants have been proposed, a systematic comparison among these variants is beyond the scope of the present study. As demonstrated in **Figure 5**, the intricate architectural design is depicted in detail.

**Figure 5:**
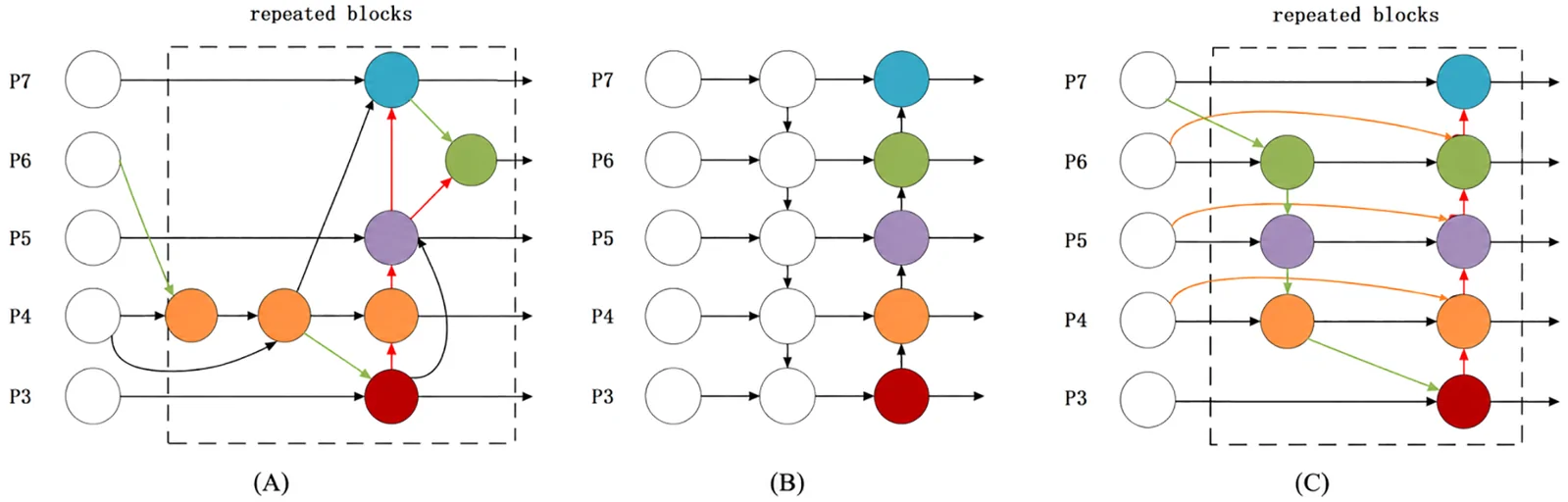
Schematic diagrams of different feature fusion structures: **(A)** NAS-FPN; **(B)** PANet; and **(C)** BiFPN.

To account for unequal contributions from features at different resolution levels, BiFPN introduces the Fast Normalized Fusion algorithm with learnable weights, replacing traditional equal-weight summation.

Taking the feature fusion at the P6 level as an example, the corresponding formulations are given in (Equations 7, 8):

(7) $$P_{6}^{td}=Conv(\frac{w_{1}\cdot P_{6}^{in}+w_{2}\cdot Resize(P_{7}^{in})}{w_{1}+w_{2}+\epsilon })$$

(8) P6out=Conv(w1'·P6in+w2'·P6td+w3'·Resize(P5out)w1'+w2'+w3'+ϵ)

In these equations, ${\text{P}}_{6}^{\text{td}}\;$denotes the top-down fused feature at the ${\text{P}}_{6}$ level, and ${\text{P}}_{6}^{\text{out}}$ represents the final output feature at the ${\text{P}}_{6}$ level after bidirectional fusion. ${\text{P}}_{6}^{\text{in}}$ and ${\text{P}}_{7}^{\text{in}}$ denote the input feature maps from the ${\text{P}}_{6}$ and ${\text{P}}_{7}$ levels, respectively, while ${\text{P}}_{5}^{\text{out}}$ denotes the output feature from the adjacent ${\text{P}}_{5}$ level. Resize(·) is used to adjust feature maps to the same spatial resolution before fusion, and Conv(·) denotes a convolution operation for feature refinement after fusion. $w_{1},w_{2},w_{1\prime },w_{2\prime },\mathrm{and}w_{3}$ are learnable scalar fusion weights, where ${\text{w}}_{\text{i}}\geq 0$. The prime symbol is used to distinguish the learnable weights in the bottom-up fusion path from those in the top-down fusion path. ϵ is a small constant introduced to avoid numerical instability during normalization.

During training, the fusion weights are jointly optimized with the other network parameters, enabling the model to learn the relative contributions of features from different pyramid levels. These learned weights are subsequently used during inference and are not regenerated according to the weather condition of each input image. Therefore, Weighted BiFPN in this study is used as a learnable weighted bidirectional multi-scale fusion mechanism rather than an input-dependent weather-adaptation mechanism. By integrating fine-grained spatial information and higher-level semantic information across multiple scales, the module improves multi-scale feature interaction within the neck network. Compared with more complex feature-pyramid structures such as NAS-FPN (Ghiasi et al., 2019), Weighted BiFPN provides a relatively simple multi-scale fusion architecture while maintaining efficient feature interaction.

#### 2.3.4 Introduction of the scale-dynamically adjusted SD-DIoU loss function

It has been demonstrated that both SCSA and BiFPN enhance feature expression capabilities; the former through trunk feature optimization and the latter through multi-scale fusion. These enhancements have been shown to mitigate interference from complex meteorological conditions to a certain extent. However, in actual orchard scenarios, edge blurring and fruit occlusion caused by foggy and rainy weather still lead to unstable geometric relationships between predicted bounding boxes and ground truth boxes during the regression phase. Traditional IoU-based losses demonstrate gradient fluctuations on samples with ambiguous boundaries or substantial scale variations. This phenomenon limits the effective conversion of front-end feature advantages into localization accuracy. To address this issue, this paper introduces the SD-DIoU loss function. The SD-DIoU loss function strengthens geometric consistency constraints during regression. Doing so enhances boundary convergence stability in complex scenarios.

Despite the evident enhancement of feature quality resulting from architectural optimization, the inherent uncertainty in annotated boundaries remains an issue. This predicament arises from the phenomenon of edge “blurring,” which is attributable to rainy and foggy conditions. This regression-level bottleneck is difficult to eliminate solely through forward feature enhancement strategies. To address this limitation, this study adopts a scale-based dynamic (SD) adjustment mechanism (Yang et al., 2025) and integrates it with the Distance-IoU (DIoU) loss (Zheng et al., 2020) to formulate the SD-DIoU loss used in this study, as defined in (Equations 9, 10):

(9) $$L_{SD-DIoU}=(1-\delta +\beta )(1-IoU+\alpha v)+(1+\delta -\beta )\frac{\rho^{2}(b,b^{gt})}{c^{2}}$$

(10) $$\beta =min(\frac{A_{gt}}{A_{ref}}\delta ,\delta )$$

where ${\text{L}}_{\text{SD}-\text{DIoU}}$ denotes the scale-dynamically adjusted bounding-box regression loss, and IoU represents the intersection over union between the predicted bounding box and the ground-truth box. b and $b^{gt}$ denote the center points of the predicted and ground-truth boxes, respectively, while $\rho^{2}(b,b^{gt})$ represents the squared Euclidean distance between the two center points. *c* denotes the diagonal length of the smallest enclosing box covering both boxes. The term v measures the aspect-ratio discrepancy between the predicted and ground-truth boxes, and α=v/(1−IoU+v+ϵ), where $\text{ϵ}=10^{-7}$. The scale-dependent factor β is calculated using (Equation 10), where ${\text{A}}_{\text{gt}}$ denotes the ground-truth bounding-box area and ${\text{A}}_{\text{ref}}$ denotes the reference area. Here, ${\text{A}}_{\text{gt}}$ is calculated from the ground-truth bounding-box dimensions used in the regression loss, and ${\text{A}}_{\text{ref}}$ is defined on the same scale, making the ratio ${\text{A}}_{\text{gt}}$/ ${\text{A}}_{\text{ref}}$ dimensionless. The reference area ${\text{A}}_{\text{ref}}$ and the adjustment parameter δ are set to 81 and 0.5, respectively. This scale-dependent weighting adjusts the relative contributions of the overlap-related and center-distance terms according to target scale.

From a regression optimization perspective, SD-DIoU complements the feature-level improvements provided by SCSA and BiFPN. For distant small-scale targets, even slight localization deviations may cause obvious IoU fluctuations (Wang et al., 2022). The adaptive weighting mechanism introduced by SD-DIoU smooths the regression optimization process, enabling the high-quality features extracted by SCSA and BiFPN to be translated into more stable convergence signals, thereby achieving coordinated improvements in detection accuracy and training stability.

### 2.4 Algorithm parameter settings and environment configuration for the experiment

The experiments were conducted on Windows 10 using Ultralytics 8.3.63, Python 3.10.0, PyTorch 2.1.1, and CUDA 11.8. The central processing unit (CPU) model employed in this experiment was the Intel Xeon W9-3475X 4.8GHz. The GPU used in this experiment was an NVIDIA Quadro RTX 8000 with 48 GB of GDDR6 memory, with 45 GB of dedicated GPU memory reported by Windows Task Manager. The hyperparameter settings used for the YOLOv12s baseline, BSS-YOLO, and ablation experiments are summarized in **Table 3**. The main single-run experiments and ablation study used random seed 0. For the repeated-run analysis, YOLOv12s and BSS-YOLO were each trained independently five times using random seeds 0, 1, 2, 3, and 4. The dataset split and all other training and evaluation settings were kept identical across the five repeated runs.

**Table 3:** Algorithmic parameter settings for the experiment.

Parameters	Setup
Epochs	200
Batch Size	32
Optimizer	SGD
Initial Learning Rate	0.01
Final Learning Rate Factor (lrf)	0.01
Momentum	0.90
Weight Decay	0.0005
Learning rate Schedule	Linear decay (cos_lr=False)
Warm-up Epochs	3
Image Size	640×640
Confidence Threshold	0.25
NMS IoU Threshold	0.70
Random Seed	0

For the YOLOv12s baseline, BSS-YOLO, and ablation configurations, SGD was used with an initial learning rate of 0.01, momentum of 0.90, and weight decay of 0.0005. Both YOLOv12s and BSS-YOLO were trained from scratch without pretrained weights. The principal online augmentation settings were HSV adjustment (h = 0.015, s = 0.70, and v = 0.40), translation (0.10), scaling (0.50), horizontal flipping (0.50), and Mosaic augmentation (1.0). Mosaic augmentation was disabled during the final 10 epochs. For the customized network configuration, one SCSA module was inserted after the backbone P5/32 feature output at layer 9, with a repeat number of 1 and an input/output channel configuration of 512/512. Five BiFPN fusion nodes were used at layers 14, 17, 20, 23, and 26, with each node repeated once. All BiFPN nodes operated on 128-channel features; the two-input nodes used a [128, 128]→128 configuration, while the three-input nodes used [128, 128, 128]→128. The final detection head used the P3/8, P4/16, and P5/32 feature levels.

### 2.5 Evaluation indicators

Object detection models necessitate the utilization of evaluation metrics to assess their performance. In order to evaluate maturity detection models, this study employs four assessment metrics, namely precision, recall, average precision ( AP), and mean average precision ( mAP). These metrics facilitate a comprehensive evaluation of maturity detection models for the honey peach maturity detection task. The calculation formulas for the relevant evaluation metrics are given in (Equations 11–14):

(11) $$Precision=\frac{TP}{TP+FP}$$

(12) $$Recall=\frac{TP}{TP+FN}$$

The term Precision in this context refers to the proportion of predictions that are true positives. This metric is a measure of the model’s ability to identify correct positives. Recall on the other hand, indicates the proportion of all true positives that are correctly detected. This metric assesses the model’s ability to identify all true positives. In this analysis, TP denotes true positives, FP denotes false positives, and FN denotes false negatives.

(13) AP=∫P(R)dR×100%

*AP* is represented by the area under the Precision-Recall curve, providing a comprehensive evaluation of a model’s detection performance across different confidence thresholds. *AP* simultaneously considers both the model’s classification capability and the accuracy of target localization. In this context, *P* denotes precision and *R* denotes recall.

(14) $$mAP=\frac{\sum_{i=1}^{n}AP_{i}}{n}$$

mAP is calculated by averaging the *AP* values across all target classes in a data-set. As the most fundamental evaluation metric in object detection tasks, this indicator reflects a model’s overall recognition capability for multiple target classes. In this equation, $AP_{i}$ denotes the average precision for the category, and n represents the total number of categories.

## 3 Results

To analyze the effects of the proposed components on model convergence, all models were trained under identical experimental settings, including the same dataset split, training strategy, hardware environment, and hyperparameters. The YOLOv12s baseline, the three single-component variants incorporating SCSA, BiFPN, or SD-DIoU, and the final BSS-YOLO model were selected for comparison. **Figure 6** presents their mAP50–95 convergence curves.

**Figure 6:**
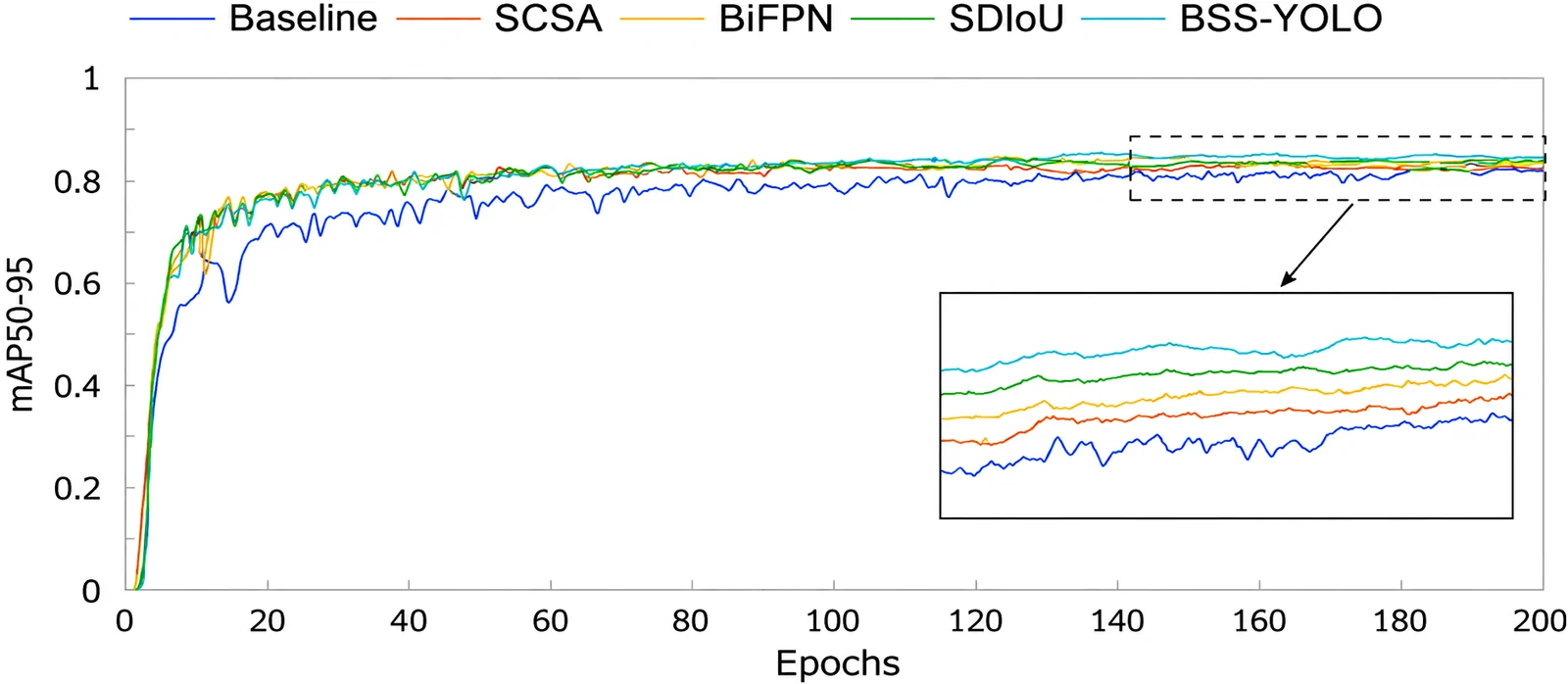
Impact of core module methods on the convergence of mAP50-95.

### 3.1 Ablation experiment

This section evaluates the effects of the proposed components by introducing them individually and in different combinations into the YOLOv12s baseline. Performance is assessed using Precision, Recall, mAP50, mAP50-95, parameter count, and inference speed.

As shown in **Table 4**, the effects of the three components are not uniformly additive across all evaluation metrics. When introduced individually, SCSA, BiFPN, and SD-DIoU increase mAP50 from 91.4% to 92.2%, 92.6%, and 92.0%, respectively, while mAP50–95 increases from 83.1% to 83.4%, 83.9%, and 84.0%. However, some two-component configurations do not consistently improve all metrics. The combination of SCSA and SD-DIoU yields an mAP50 of 91.6% and a Recall of 83.8%, while BiFPN combined with SD-DIoU results in a Recall of 81.9%. Similarly, the combination of SCSA and BiFPN obtains an mAP50 of 91.4%, equal to that of the baseline, although its mAP50–95 increases to 84.5%. These results indicate that the effects of the components are not simply additive, and different combinations affect the evaluation metrics differently. When all three components are combined, the model achieves 89.8% Precision, 87.8% Recall, 93.4% mAP50, and 85.0% mAP50-95. Among the evaluated ablation configurations, the complete model achieves the highest mAP50 and mAP50-95.

**Table 4:** Ablation experiments.

Baseline	SCSA	BiFPN	SD-DIoU	GFLOPs	Precision (%)	Recall (%)	mAP50 (%)	mAP50-95 (%)	Params (M)	FPS
✓				19.3	88.1	85.2	91.4	83.1	9.07	88.5
✓	✓			21.2	86.8	87.5	92.2	83.4	9.20	72.4
✓		✓		21.9	86.8	87.6	92.6	83.9	8.56	72.0
✓			✓	19.3	90.1	86.1	92.0	84.0	9.07	88.3
✓	✓		✓	21.2	90.9	83.8	91.6	83.7	9.20	73.2
✓		✓	✓	21.9	89.6	81.9	92.8	83.8	8.56	71.0
✓	✓	✓		21.9	89.2	84.1	91.4	84.5	8.57	64.4
✓	✓	✓	✓	21.9	89.8	87.8	93.4	85.0	8.57	63.5

Baseline is YOLOv12s. The label √ indicates the method(s) used.

This behavior may be related to the different functional stages affected by the three components. SCSA modifies spatial–channel feature representation in the backbone, whereas BiFPN subsequently performs learnable weighted fusion of features from different pyramid levels in the neck. Therefore, changes introduced by SCSA at the feature-representation stage may alter the information subsequently received by the multi-scale fusion network, while BiFPN determines how features from different pyramid levels contribute to the fused representation. When either component is used independently, such changes do not necessarily lead to simultaneous improvements in Precision, Recall, and mAP. When SCSA and BiFPN are combined, the refined backbone features are further integrated across pyramid levels, which may partly explain the recovery of Precision observed in this configuration. However, the ablation results are interpreted as configuration-dependent interactions rather than evidence of a strictly additive or universally synergistic effect. SD-DIoU further affects bounding-box regression optimization at a different stage, and the complete configuration ultimately achieves the highest mAP50 and mAP50–95 among the evaluated combinations. The ablation results in **Table 4** were obtained using the fixed random seed adopted for the main experiments. Accordingly, small differences among intermediate module combinations are interpreted descriptively rather than as evidence of statistically stable improvements.

### 3.2 Comparison experiments

#### 3.2.1 Comparative experiments of different models

During the baseline model selection phase, we compared multiple YOLO-series models and other representative object detectors in terms of detection accuracy, computational complexity, and inference efficiency. Experimental results are presented in **Table 5**.

**Table 5:** Comparison experiments.

Model	GFLOPs	Precision (%)	Recall (%)	mAP50 (%)	mAP50-95 (%)	Params (M)	FPS
YOLOv5s	23.8	90.0	83.9	90.2	82.3	9.11	168.1
YOLOv8s	28.4	84.2	84.4	90.4	81.4	11.13	166.2
YOLOv9s	26.7	89.5	84.2	91.6	82.5	7.17	63.3
YOLOv11s	21.3	88.5	86.3	91.1	82.9	9.08	88.1
YOLOv12s	19.3	88.1	85.2	91.4	83.1	9.07	88.5
YOLOv13s	20.7	86.2	86.0	91.7	82.9	9.02	63.7
RT -DETR-l	103.4	86.6	80.5	82.4	74.0	31.99	32.6
RT -DETR-x	232.0	91.2	82.6	84.3	75.4	67.31	22.4
EMA -YOLO	17.6	86.1	79.1	89.5	80.4	12.80	106.1
BSS-YOLO(ours)	21.9	89.8	87.8	93.4	85.0	8.57	63.5

FPS represents model forward-pass throughput measured with batch size = 1 and FP32 inference, excluding preprocessing and NMS.

To improve the comparability of the evaluated models, all compared models were retrained locally using the same dataset split, training input resolution, and number of training epochs. All models were trained from scratch without pretrained weights and were evaluated using the same test set, evaluation protocol, and hardware platform. The fixed-seed results reported in **Table 5** were obtained using random seed 0.

Optimization-related settings, including the optimizer, learning-rate schedule, warm-up strategy, and associated hyperparameters, followed the recommendations of the corresponding official implementations and were therefore not necessarily identical across model families. This choice was intended to evaluate each detector under its representative training configuration and to avoid disadvantaging individual architectures by applying a single optimization configuration without model-specific retuning. Consequently, the results in **Table 5** should be interpreted as end-to-end comparisons under model-specific recommended training configurations rather than as a strictly architecture-controlled comparison.

For the central comparison between YOLOv12s and BSS-YOLO, the optimizer, learning-rate schedule, warm-up strategy, data augmentation, training budget, and evaluation settings were kept identical. Therefore, the performance difference between YOLOv12s and BSS-YOLO was evaluated under a directly controlled training protocol. The stability of this improvement across five independent runs is further evaluated in Section 3.2.2.

Inference efficiency was evaluated using the same forward-pass benchmarking procedure for all models. The batch size was set to 1 and the input resolution to 640 × 640. Before timing, 200 inference forward passes were performed as runtime warm-up, followed by 1,000 timed forward passes. CUDA synchronization was performed immediately before and after each timed forward pass. The benchmark used FP32 inference and excluded image loading, preprocessing, and post-processing/NMS. Therefore, the reported FPS values represent model forward-pass throughput rather than end-to-end application speed.

As shown in **Table 5**, different models exhibit distinct advantages. Specifically, compared with the widely adopted YOLOv8s, YOLOv12s achieves an mAP50–95 that is 1.7 percentage points higher (83.1% vs. 81.4%) while reducing the computational cost. Its GFLOPs and parameter count are reduced by 32.0% and 18.5%, respectively. Furthermore, when evaluated against recent models such as YOLOv11s and YOLOv13s, YOLOv12s demonstrates a favorable trade-off between detection accuracy and computational efficiency. Although YOLOv13s obtains a slightly higher mAP50, YOLOv12s achieves a higher mAP50–95 with fewer GFLOPs, indicating stronger performance across stricter IoU thresholds. In addition, YOLOv12s requires 9.4% and 6.8% fewer GFLOPs than YOLOv11s and YOLOv13s, respectively, while its forward-pass FPS is also higher than that of YOLOv13s under the benchmark protocol used in this study. Therefore, YOLOv12s was selected as the baseline model for subsequent improvements.

In the fixed-seed comparison reported in **Table 5**, BSS-YOLO achieves the highest mAP50 and mAP50–95 values among the compared models. Compared with the YOLOv12s baseline under identical training and evaluation settings, BSS-YOLO improves Precision, Recall, mAP50, and mAP50–95 by 1.7, 2.6, 2.0, and 1.9 percentage points, respectively, while reducing the parameter count from 9.07M to 8.57M. Compared with the recent peach-related EMA-YOLO model, BSS-YOLO improves mAP50 and mAP50–95 by 3.9 and 4.6 percentage points, respectively, showing higher detection accuracy under the evaluated conditions. Although EMA-YOLO requires fewer GFLOPs and achieves a higher inference speed, BSS-YOLO achieves higher Recall, mAP50, and mAP50–95 while maintaining a smaller parameter count. In addition, the RT-DETR variants require substantially greater computational resources, and their inference speeds are lower than that of BSS-YOLO. For example, RT-DETR-l requires 103.4 GFLOPs and 31.99M parameters, whereas BSS-YOLO requires 21.9 GFLOPs and 8.57M parameters. These results indicate that BSS-YOLO maintains a competitive balance between detection accuracy, model complexity, and forward-pass inference efficiency under the model-specific training configurations and unified inference benchmark adopted in this study.

#### 3.2.2 Repeated-run performance analysis

To assess run-to-run variability, YOLOv12s and BSS-YOLO were each trained independently five times using random seeds 0, 1, 2, 3, and 4. Within each model, the dataset split and all training settings were kept unchanged across runs. The mean and sample standard deviation of Precision, Recall, mAP50, and mAP50–95 are reported in **Table 6**.

**Table 6:** Performance of YOLOv12s and BSS-YOLO over five independent training runs.

Model	Precision (%)	Recall (%)	mAP50 (%)	mAP50-95 (%)
YOLOv12s	88.48 ± 0.69	84.86 ± 0.56	91.52 ± 0.30	83.14 ± 0.26
BSS-YOLO	89.98 ± 0.38	86.16 ± 1.37	93.30 ± 0.14	84.82 ± 0.24

Values are reported as the mean ± sample standard deviation over five independent training runs using random seeds 0–4.

Across five independent runs, BSS-YOLO achieved 93.30 ± 0.14% mAP50 and 84.82 ± 0.24% mAP50-95, whereas YOLOv12s achieved 91.52 ± 0.30% and 83.14 ± 0.26%, respectively. Thus, BSS-YOLO improved the mean mAP50 and mAP50–95 by 1.78 and 1.68 percentage points, respectively. BSS-YOLO also achieved higher mAP50–95 than YOLOv12s under all five matched random seeds. The per-seed improvements ranged from 1.3 to 2.1 percentage points, with a mean paired improvement of 1.68 ± 0.33 percentage points.

### 3.3 Performance analysis under different conditions

#### 3.3.1 Class-wise performance

To provide a more detailed evaluation of ripeness recognition performance, class-wise metrics were extracted from the evaluation of BSS-YOLO on the complete 600-image test set. The Precision, Recall, mAP50, and mAP50–95 values for the unripe (UR), half-ripe (HR), and ripe (R) classes are reported in **Table 7**.

**Table 7:** Class-wise performance of BSS-YOLO on the complete test set.

Ripeness class	Precision (%)	Recall (%)	mAP50 (%)	mAP50-95 (%)
UR	86.5	82.1	88.9	76.9
HR	90.9	87.4	94.5	88.0
R	92.1	94.0	96.7	90.2

As shown in **Table 7**, the R class achieved mAP50 and mAP50–95 values of 96.7% and 90.2%, respectively, followed by the HR class with 94.5% and 88.0%. The corresponding values for the UR class were 88.9% and 76.9%. The R class also achieved the highest Precision and Recall, whereas the UR class had the lowest values across all four metrics.

The normalized confusion matrix in **Figure 7** further shows that the predictions are predominantly concentrated along the main diagonal. The normalized diagonal proportions for UR, HR, and R are approximately 0.85, 0.85, and 0.93, respectively. The remaining cross-class confusion is mainly observed between adjacent ripeness stages, particularly between UR and HR, which is consistent with the gradual visual transition in peel-color characteristics between neighboring ripeness categories.

**Figure 7:**
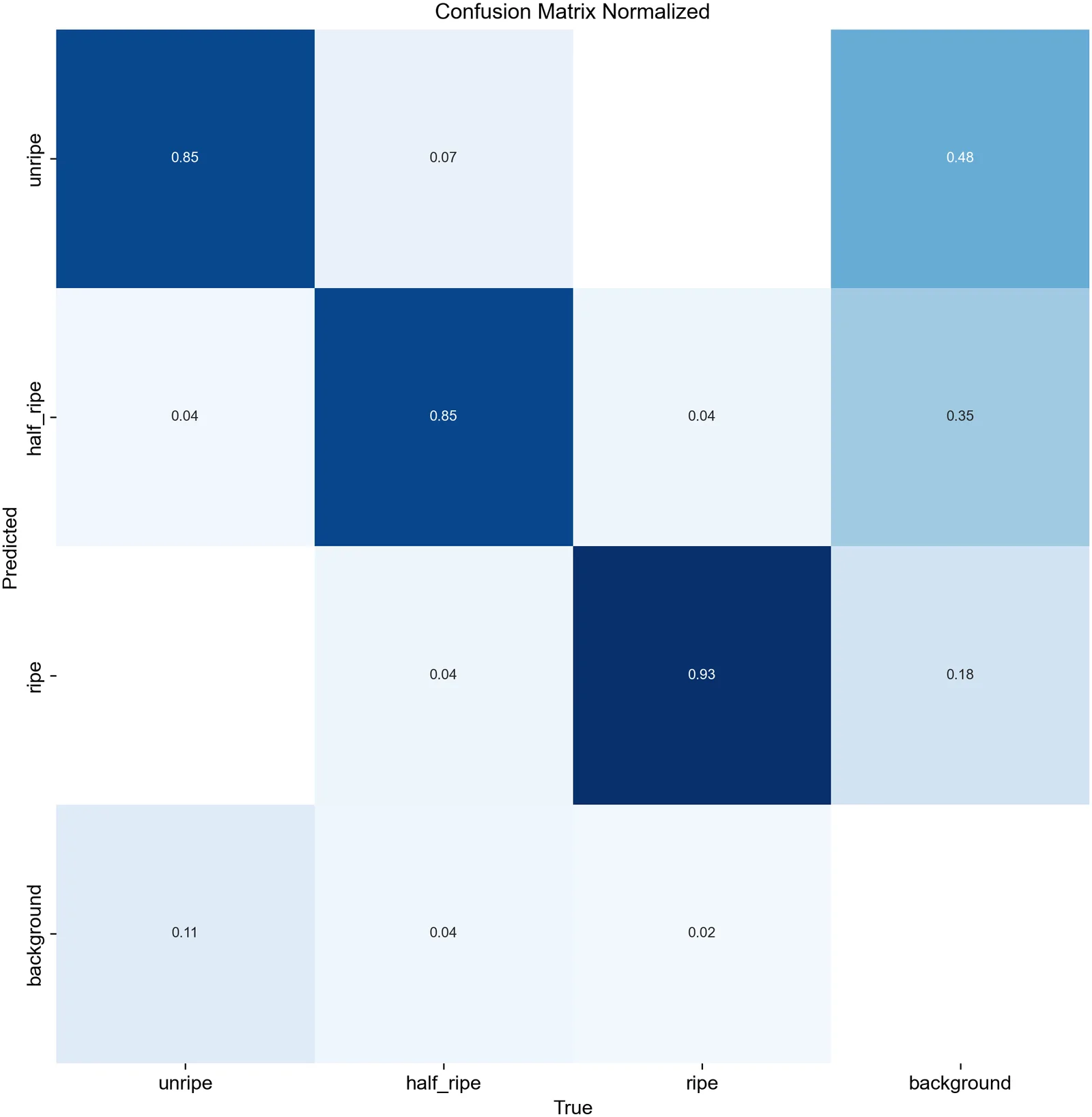
Normalized confusion matrix of BSS-YOLO on the complete test set.

#### 3.3.2 Weather-condition analysis

To evaluate the influence of weather-related image degradation, the final BSS-YOLO model was evaluated separately on three subsets of the test set: the field-collected sunny subset, the corresponding simulated fog subset, and the simulated rain subset. Each condition-specific subset contained 200 images and 526 annotated targets. In addition, the complete test set containing 600 images and 1,578 annotated targets was evaluated under the same model and evaluation protocol. The corresponding results are presented in **Table 8**.

**Table 8:** Detection performance of BSS-YOLO on the field-collected sunny test subset, simulated weather-degraded subsets, and the complete test set.

Weather condition	Precision (%)	Recall (%)	mAP50 (%)	mAP50-95 (%)
Field-collected sunny	89.9	88.1	93.9	85.9
Simulated fog	88.1	88.4	93.0	84.3
Simulated rain	91.0	88.0	93.5	85.1
Overall test	89.8	87.8	93.4	85.0

As shown in **Table 8**, BSS-YOLO achieved mAP50 values of 93.9%, 93.0%, and 93.5% on the field-collected sunny, simulated fog, and simulated rain subsets, respectively, with corresponding mAP50–95 values of 85.9%, 84.3%, and 85.1%. Compared with the field-collected sunny subset, simulated fog reduced mAP50 and mAP50–95 by 0.9 and 1.6 percentage points, respectively, whereas simulated rain resulted in smaller decreases of 0.4 and 0.8 percentage points. These results indicate that simulated fog produced a more noticeable performance degradation than simulated rain under the evaluated conditions.

On the complete 600-image test set, BSS-YOLO achieved 89.8% Precision, 87.8% Recall, 93.4% mAP50, and 85.0% mAP50-95. Because the three condition-specific subsets were evaluated independently, the overall test-set metrics were calculated jointly on the complete test set and are not the arithmetic averages of the three subset-level results.

The field-collected sunny subset provides quantitative evaluation on real orchard images under the evaluated sunny conditions. In contrast, the fog and rain subsets are simulation-based; therefore, their results are interpreted only within the simulated adverse-weather conditions considered in this study and are not regarded as evidence of general robustness under naturally occurring fog or rainfall.

#### 3.3.3 Occlusion-level analysis

To evaluate the effect of occlusion on detection performance, the ground-truth targets in the test set were manually grouped into two occlusion levels according to their visible degree of occlusion. Low occlusion refers to targets with little or no obvious occlusion, whereas Moderate-to-high occlusion refers to targets with more noticeable occlusion that remained within the original valid annotation range. This grouping was used only for the subgroup evaluation of the test set. The quantitative results for the two occlusion levels are summarized in **Table 9**.

**Table 9:** Detection performance of BSS-YOLO under different occlusion levels.

Occlusion level	Number of targets	Correct detection	Recall (%)
Low	936	870	92.95
Moderate-to-high	642	516	80.37

The Recall decreased from 92.95% under Low occlusion to 80.37% under Moderate-to-high occlusion, corresponding to a decrease of 12.58 percentage points. This result indicates that increasing occlusion has a clear adverse effect on detection performance. According to the original annotation protocol, targets with more than 75% occlusion were excluded from annotation and therefore fall outside the quantitative evaluation range of the present study.

#### 3.3.4 Target-size analysis

Model performance was also analyzed according to target size. Based on the bounding-box area at the 640 × 640 input resolution, the targets were divided into Small (<32²), Medium (32²–96²), and Large (≥96²) groups. The complete 600-image test set was used for this analysis. The quantitative results for the three target-size groups are summarized in **Table 10**.

**Table 10:** Detection performance of BSS-YOLO for different target sizes.

Target size	Number of targets	Correct detection	Recall (%)
Small	75	47	62.67
Medium	447	359	80.31
Large	1056	980	92.80

The Recall increased from 62.67% for Small targets to 80.31% for Medium targets and 92.80% for large targets. These results show a clear scale-dependent trend under the evaluated conditions. BSS-YOLO performs more reliably on medium and large targets, whereas small-target detection remains comparatively challenging.

Overall, these supplementary analyses provide a more detailed characterization of BSS-YOLO beyond the overall mAP, including its performance across ripeness categories, simulated weather conditions, occlusion levels, and target scales.

### 3.4 Visual and interpretability analysis

This section offers an examination of the model’s detection behavior that extends beyond quantitative experimentation, providing a qualitative analysis supported by visual evidence. Through the presentation of detection outcomes and heatmap distributions, the model’s experimental results under diverse environmental conditions are elucidated.

#### 3.4.1 Visual analysis of detection results

To provide an intuitive qualitative comparison of detection behavior under the evaluated weather conditions, one orchard scene containing peach targets with different apparent sizes and partial occlusion was selected for visualization. The same source image was used to construct the sunny, simulated-fog, and simulated-rain inputs, and identical inputs were evaluated by YOLOv12s and BSS-YOLO, as shown in **Figure 8**. The three columns from left to right correspond to sunny, simulated fog, and simulated rain conditions, respectively, while the rows from top to bottom show the input images, the detection results of YOLOv12s, and the detection results of the proposed BSS-YOLO model.

**Figure 8:**
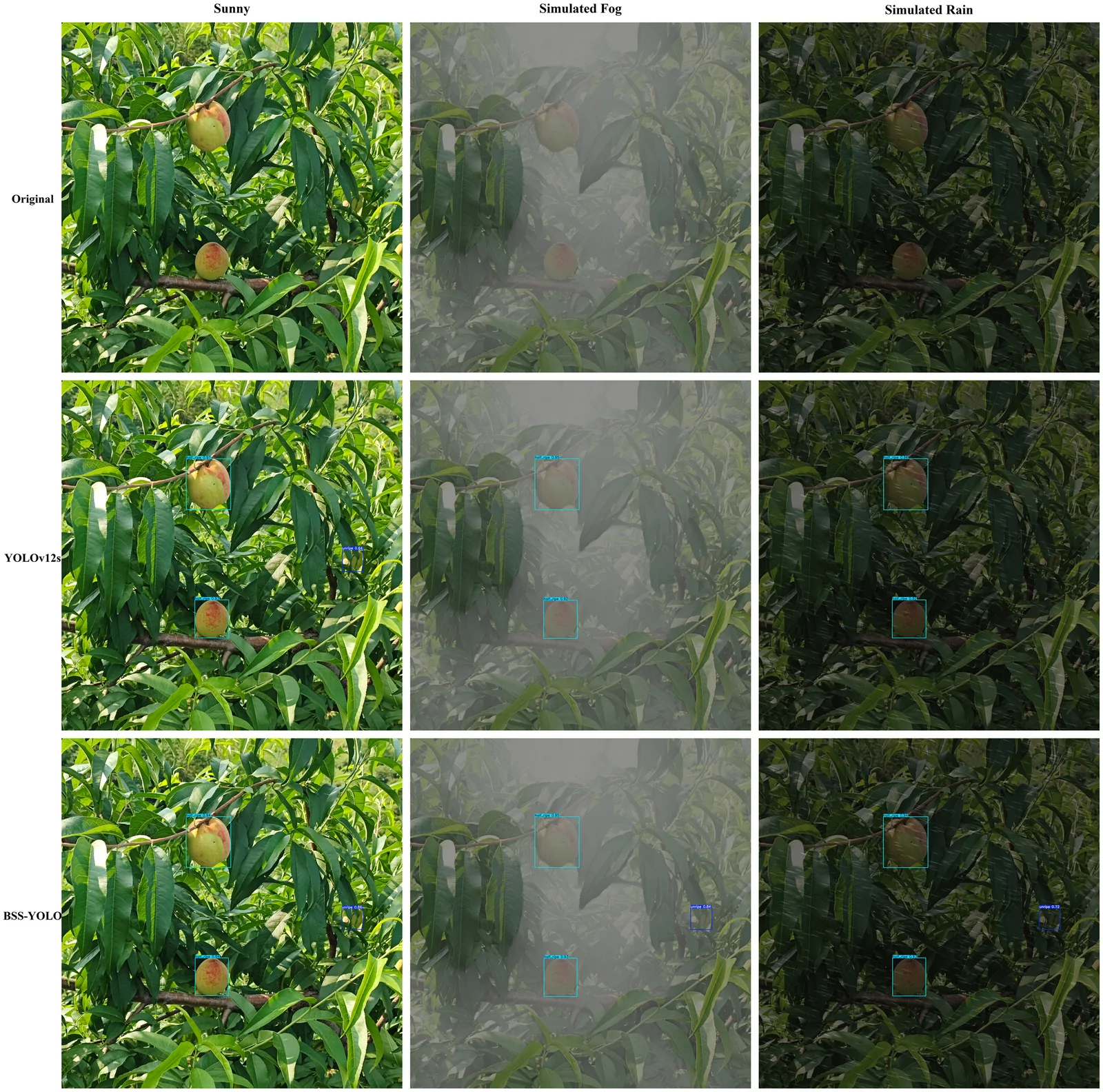
Qualitative detection comparison between YOLOv12s and BSS-YOLO under sunny, simulated-fog, and simulated-rain conditions.

As illustrated in **Figure 8**, both YOLOv12s and BSS-YOLO detect the two clearly visible half-ripe fruits under the three evaluated conditions. For the smaller and partially occluded unripe fruit on the right side of the image, YOLOv12s detects the target under sunny conditions but does not detect it in the simulated fog and rain examples, whereas BSS-YOLO retains a detection in these two degraded versions. This observation is limited to the displayed example and is used only to qualitatively illustrate the detection behavior of the two models under the evaluated conditions. The assessment of overall detection performance is based primarily on the quantitative results reported above.

#### 3.4.2 Qualitative visualization of attention heat-maps

To intuitively illustrate the differences in model attention under the evaluated meteorological conditions, attention heat-maps under sunny, simulated-fog, and simulated-rain conditions are visually compared in **Figure 9**. Grad-CAM++ was used to generate the heat-map for both YOLOv12s and BSS-YOLO. For each model, the three multi-scale feature layers (P3, P4, and P5) used as inputs to the Detect head were selected for visualization; although their layer indices differ because of the different network architectures, the selected layers occupy equivalent functional positions in the detection pipeline. The CAM responses were re-normalized within the detected bounding-box regions and displayed using the same normalization and visualization procedure. Each heat-map was independently normalized to the [0, 1] display range; therefore, the heat-map intensity is interpreted qualitatively rather than as a quantitative comparison of absolute activation magnitude across models. The resulting visualizations show that, in the displayed examples, the response regions of BSS-YOLO remain concentrated around the peach targets under the evaluated conditions. These heat-maps are used as supplementary qualitative interpretation of the quantitative detection results rather than as independent evidence of model robustness.

**Figure 9:**
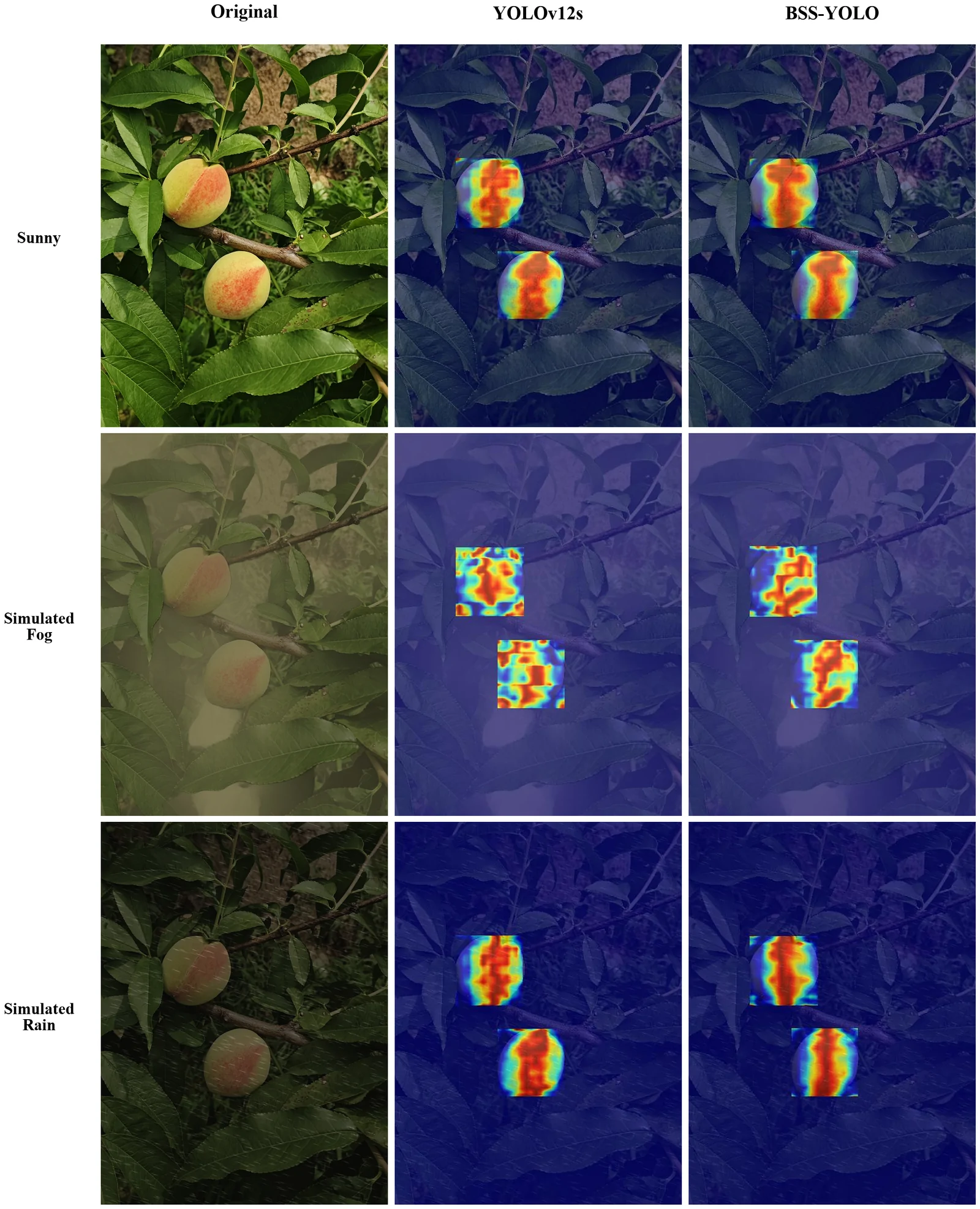
Grad-CAM++ heat-map visualization under sunny, simulated fog, and simulated rain conditions.

To provide a more balanced qualitative assessment, **Figure 10** presents a challenging example associated with a missed detection. The corresponding Grad-CAM++ visualization shows weak or absent activation around the missed target. This example is included to illustrate a difficult detection case under the evaluated conditions and to complement the preceding qualitative analysis.

**Figure 10:**
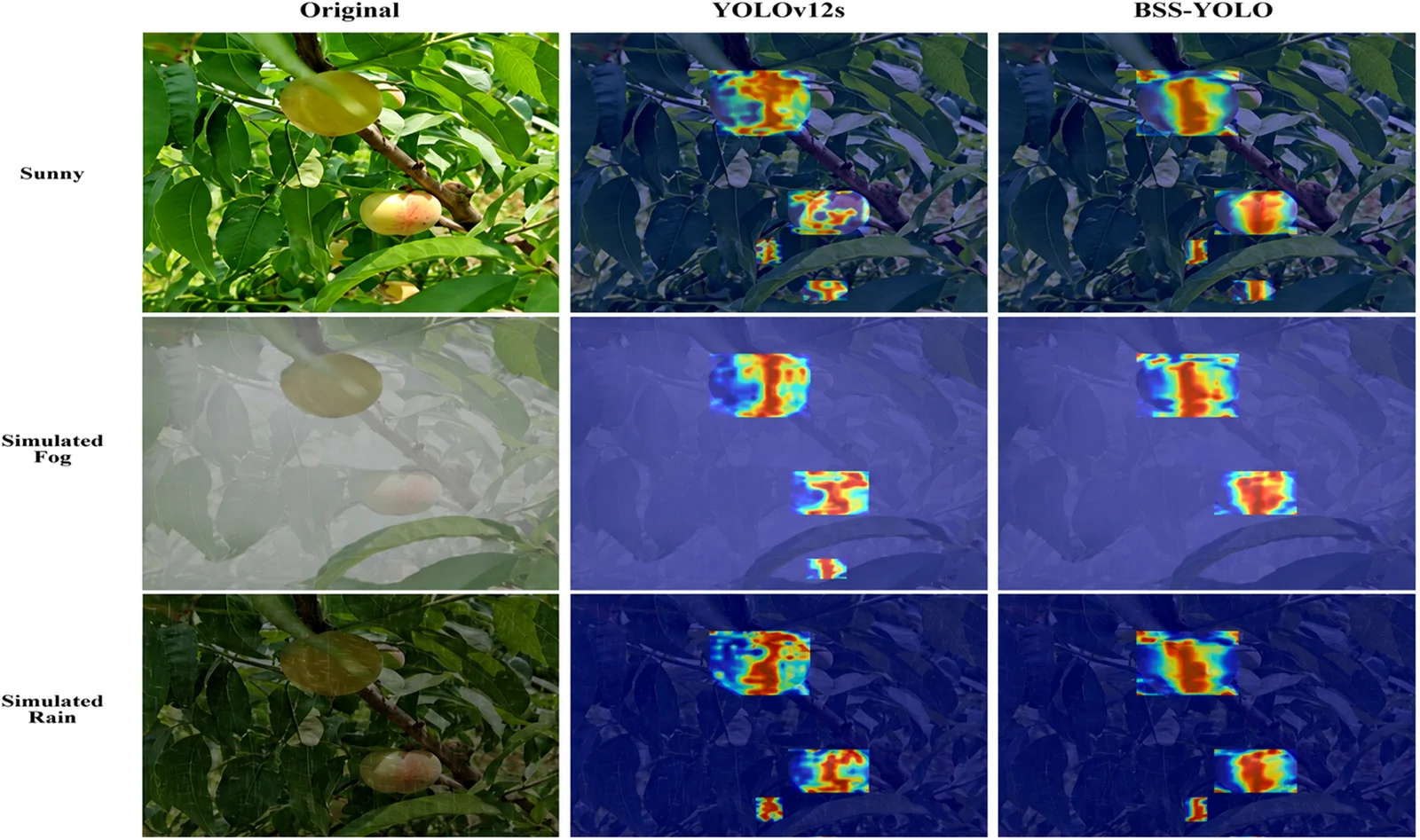
Grad-CAM++ heat-map for a challenging case involving a missed detection.

## 4 Discussion

### 4.1 Effect of physics-inspired meteorological augmentation

The physics-inspired augmentation strategy was designed to introduce controllable fog- and rain-related image degradation through atmospheric attenuation, scattering, and synthetic rain effects, rather than relying solely on arbitrary pixel-level perturbations. Under the simulated adverse-weather conditions used in this study, the resulting degradation patterns provide a more structured form of meteorological disturbance for model training and evaluation.

The experimental results under the constructed simulated-weather setting suggest that the use of physics-inspired degradation can help the model maintain relatively stable detection performance across the evaluated sunny, simulated fog, and simulated rain conditions. This observation is consistent with the use of degradation processes that preserve certain physical characteristics of weather-related image formation. Accordingly, the role of the augmentation strategy in this study is interpreted as improving adaptation to the simulated meteorological degradation conditions considered in the experiments.

Because the fog and rain images used in this study are simulation-based, differences from naturally occurring adverse-weather scenes may remain. Therefore, the corresponding results are interpreted within the simulated meteorological degradation conditions evaluated in this study rather than as general validation under real adverse-weather conditions.

### 4.2 Interaction between SCSA and BiFPN

The ablation results show that SCSA and BiFPN do not produce uniform improvements across all evaluation metrics when introduced individually or in combination. When SCSA is introduced alone, Recall and mAP50 increase relative to the baseline, whereas Precision decreases. A similar decrease in Precision is observed when BiFPN is introduced alone. When SCSA and BiFPN are combined, Precision increases to 89.2%, while Recall decreases to 84.1% and mAP50 remains at 91.4%, equal to that of the baseline. Therefore, their combined effect should not be interpreted as a simple additive or uniformly synergistic improvement.

From the architectural perspective, SCSA mainly modifies spatial-channel feature representation in the backbone, whereas BiFPN performs learnable weighted multi-scale feature fusion in the neck. Because the two components act at different stages of the detection pipeline, their combination may affect different evaluation metrics in different ways. With the further inclusion of SD-DIoU at the regression stage, the complete configuration achieves the highest mAP50 and mAP50–95 among the evaluated ablation configurations.

### 4.3 Calibration of SD-DIoU for boundary regression

Under simulated fog and rain degradation, fruit boundaries may become less distinct, increasing the difficulty of bounding-box regression. As shown in the ablation results, introducing SD-DIoU increases mAP50–95 from 83.1% to 84.0%. This improvement is consistent with the scale-aware regression formulation of SD-DIoU, which adjusts the regression process according to target-scale information. The result suggests that SD-DIoU can provide a modest improvement in localization performance under the evaluated conditions.

### 4.4 Suggestions for further research

The cross-model benchmark used model-specific optimization settings from the corresponding official implementations. Therefore, it reflects end-to-end performance under representative training configurations rather than a strictly architecture-controlled comparison. However, YOLOv12s and BSS-YOLO were trained using identical optimization and evaluation settings, providing a directly controlled assessment of the proposed architectural modifications. The ablation study (**Table 4**) shows that the forward-pass FPS decreases from 88.5 to 63.5 after the introduction of the complete set of modules. This indicates a trade-off between improved detection accuracy and the additional computational cost introduced by the modified architecture. For computationally constrained embedded or edge platforms, actual inference efficiency may also be influenced by hardware-specific factors, such as memory bandwidth and inference-engine support.

In addition, the current study focuses on static-image detection. Model compression, reparameterization, or quantization may be considered in future work to further reduce deployment cost. Hardware-oriented acceleration strategies, such as FP16/INT8 inference and TensorRT acceleration on supported platforms, may also provide potential pathways for improving deployment efficiency.

The proposed method performs image-based visual ripeness classification rather than direct determination of the optimal harvest window. Because physiological indicators such as soluble solids content and firmness were not included in the present study, the predicted ripeness stages are used only as auxiliary visual information for harvest-related decision-making and should not be interpreted as an independent measure of physiological maturity or optimal harvest timing.

## 5 Conclusions

To address honey peach ripeness detection under complex orchard imagery and controlled weather-related degradation, this study proposes BSS-YOLO, an improved YOLOv12s-based model evaluated using field-collected sunny orchard images and physics-simulated fog and rain images. The model incorporates meteorological degradation augmentation, spatial–channel feature enhancement, weighted multi-scale feature fusion, and scale-dynamic boundary regression optimization.

Experimental results under the constructed multi-meteorological dataset show that the model maintains competitive detection performance across the evaluated sunny, simulated fog, and simulated rain conditions. The physics-inspired augmentation strategy provides controllable weather-related degradation samples for model training and evaluation. On this basis, the introduction of SCSA and BiFPN enhances ripeness-related feature representation and adaptive multi-scale feature fusion, improving target recognition in complex backgrounds, occluding regions, and overlapping fruit scenarios. In addition, the SD-DIoU loss function improves localization stability for multi-scale targets, contributing to a 0.9% increase in mAP50-95.

Comprehensive ablation and comparative experiments demonstrate that BSS-YOLO achieves 93.4% mAP50 and 85.0% mAP50–95 on the self-built multi-meteorological honey peach dataset, with only 8.57 M parameters and an inference speed of 63.5 FPS. These results indicate that the proposed method achieves a reasonable balance between detection accuracy and inference efficiency under the evaluated conditions. Overall, this study provides a honey peach ripeness detection approach that combines meteorological degradation modeling, feature enhancement, and scale-aware localization optimization, and demonstrates its effectiveness under the simulated adverse-weather conditions evaluated in this study. Due to the limitations of the current experimental conditions, future work will further validate the proposed method using field-collected orchard images under naturally occurring foggy and rainy conditions to assess its generalization performance in practical environments.

## Data Availability

The raw data supporting the conclusions of this article will be made available by the authors, without undue reservation.
